# Aortic size distribution among normal, hypertension, bicuspid, and Marfan populations

**DOI:** 10.1093/ehjimp/qyad019

**Published:** 2023-08-30

**Authors:** Jinlin Wu, Weiyue Zeng, Xiaoshan Li, Jiade Zhu, Chenyu Zhou, Ruixin Fan, Tucheng Sun, Hongwen Fei, Xin Li

**Affiliations:** Department of Cardiac Surgery, Guangdong Cardiovascular Institute, Guangdong Provincial People’s Hospital (Guangdong Academy of Medical Sciences), Southern Medical University, Guangzhou 510080, China; Department of Emergency, Guangdong Provincial People’s Hospital (Guangdong Academy of Medical Sciences), Southern Medical University, Zhongshan 2nd Rd 106, Guangzhou 510080, China; Department of Echocardiography, Guangdong Provincial People’s Hospital (Guangdong Academy of Medical Sciences), Southern Medical University, Zhongshan 2nd Rd 106, Guangzhou 510080, China; Department of Cardiac Surgery, Guangdong Cardiovascular Institute, Guangdong Provincial People’s Hospital (Guangdong Academy of Medical Sciences), Southern Medical University, Guangzhou 510080, China; Department of Cardiac Surgery, Fuwai Hospital, Beijing 100037, China; Department of Cardiac Surgery, Guangdong Cardiovascular Institute, Guangdong Provincial People’s Hospital (Guangdong Academy of Medical Sciences), Southern Medical University, Guangzhou 510080, China; Department of Cardiac Surgery, Guangdong Cardiovascular Institute, Guangdong Provincial People’s Hospital (Guangdong Academy of Medical Sciences), Southern Medical University, Guangzhou 510080, China; Department of Echocardiography, Guangdong Provincial People’s Hospital (Guangdong Academy of Medical Sciences), Southern Medical University, Zhongshan 2nd Rd 106, Guangzhou 510080, China; Department of Emergency, Guangdong Provincial People’s Hospital (Guangdong Academy of Medical Sciences), Southern Medical University, Zhongshan 2nd Rd 106, Guangzhou 510080, China

**Keywords:** thoracic aorta, aorta, dilation, aneurysm, diameter

## Abstract

**Aims:**

Large-scale investigations on ascending aortic diameter, especially in the Asian population, are lacking. Furthermore, relevant evidence regarding the distribution of hypertension (HP), bicuspid aortic valve (BAV), and Marfan syndrome (MFS) is scarce. We aimed to examine the distribution of ascending aortic diameter in these populations in China.

**Methods and results:**

The data of a total number of 698 795 individuals who underwent cardiac ultrasound were subjected to retrospective analysis. After screening, 647 087 individuals were included in the final analysis. In the normal population, the mean ascending aortic diameter was 28.1 ± 3.2 mm (27.2 ± 3.1 mm in women vs. 29.0 ± 3.1 mm in men) (*P* < 0.001). Aortic diameter increased gradually with age (*P* < 0.001). The prevalence of aortic dilation, aneurysm, and dissection in individuals with HP was 12.83%, 2.70%, and 4.77%, respectively. In individuals with MFS, the corresponding rates were 43.92%, 35.31%, and 26.11%. Notably, although BAV patients had high incidences of aortic dilation (37.00%) and aortic aneurysm (16.46%), the incidence of aortic dissection was relatively low (0.74%). Most cases of aortic dissection occurred at an aortic diameter of less than 55 mm. However, in the overall population, the incidence of aortic dissection significantly increased with the increase in the aortic diameter, revealing the existence of an ‘aortic paradox’.

**Conclusions:**

(i) The ascending diameter increases with age and is larger in men than in women; (ii) ‘Aortic paradox’ is explained; (iii) BAV bears a high rate of aortic dilation, but a low incidence of aortic dissection.

## Graphical Abstract

**Graphical Abstract:**
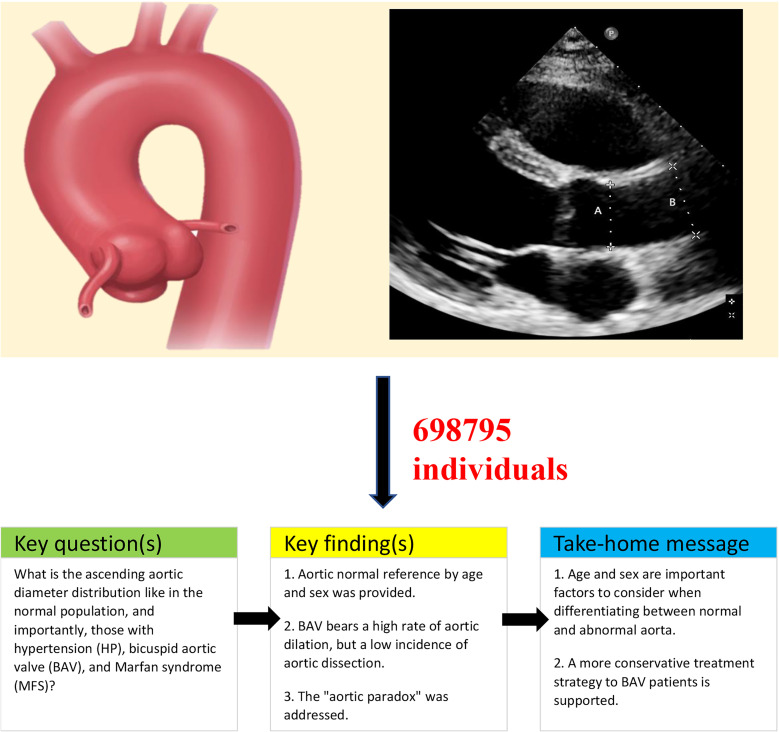

## Introduction

Aortic diseases are a high-risk group, represented mainly by thoracic aortic aneurysm (TAA), with poor prognosis in cases of adverse aortic events (AAE) such as aortic dissection or rupture. Although there are many predictors of AAE, such as wall shear stress, gene,^1^ and ascending aortic length as we have previously proposed,^2^ aortic diameter is still the most pivotal biomarker to assess the AAE risk. This clinical parameter is the key surgical indication due to its simplicity, availability, and Laplace’s law endorsement. Large-sample data for the normal aortic diameter have been rarely reported; moreover, the focus of the scarce currently available literature has been placed mainly on the Western population.^3^

Furthermore, the aortic diameter distribution in patients considered to be particularly predisposed to AAE, such as those with hypertension (HP), bicuspid aortic valve (BAV), and Marfan syndrome (MFS), is even more poorly documented. Investigations of the aortic diameter distribution among these subgroups may be helpful for the establishment of a more specified clinical management protocol. In addition, patients with these conditions are at increased risk of rapid aortic dilation. Nevertheless, whether and how much their aortic diameter distribution differs from that of the normal population requires further exploration. Importantly, the aortic diameter distribution of aortic dissection among the normal population and these aforementioned subgroups is unknown. Previous research has been focused either solely on aortic dissection (numerator) or the normal population (denominator). However, neither of them has been supposed to provide a complete reflection of the whole picture.

In this work, we aimed to investigate the distribution of ascending aortic diameters in the normal population based on age and gender. In addition, the diameter distribution in patients with HP, BAV, and MFS was also established, analysed, and compared with that of the normal population.

## Methods

This study was approved by the Institutional Review Board of Guangdong Provincial People’s Hospital (Reference Number: 2019-842H-1) and is reported in compliance with the Strengthening the Reporting of Observational Studies in Epidemiology (STROBE) guideline.^4^ The funders had no role in study design, data collection, data analysis, data interpretation, and the writing of the report. The corresponding authors had full access to all the data in the study and had final responsibility for the decision to submit for publication.

The data of a total number of 698 795 patients who underwent cardiac ultrasound at Guangdong Provincial People’s Hospital from January 2013 to December 2021 were retrospectively included in this study, and 647 087 cases were finally included after screening. The following pre-defined exclusion criteria were applied: (i) non-cardiac ultrasound (e.g. peripheral vascular ultrasound); (ii) patients aged less than 18 years; (iii) history of ascending aortic repair surgery; and (iv) absence of the data on the ascending aortic diameter. The detailed data of the screening process are presented in Supplementary data online, *Figure S1*. The ‘normal population’ is defined as those with cardiac ultrasound not suggestive of cardiovascular malformations or pathological changes, and combined with no related cardiovascular diseases. Hypertensive patients were diagnosed according to a self-reported medical history of HP. BAV was diagnosed by ultrasound morphologically. MFS was diagnosed in line with the Ghent criteria.

Standard two-dimensional (2D) echocardiography was performed according to American Society of Echocardiography (ASE) guidelines.^5^ All transthoracic 2D-echocardiograms were performed by experienced operators using ultrasound machines: (Philips Medical Systems, Andover, MA, USA) with an S5-1 probe 2.5–3.5 MHz and the GE Vivid series (GE Vingmed Ultrasound AS, Horten, Norway) with an M5S probe (2–4 MHz). Patients were placed in the left lateral decubitus position to obtain a parasternal long-axis view of the left ventricle (LV), which enabled aortic root and proximal ascending aorta visualization and subsequent measurements. The aortic annular diameter, aortic sinus diameter, and ascending aortic diameter were measured at the end-diastole. The maximum value was taken as the diameter of the aorta. Other ultrasound parameters, including the left ventricular ejection fraction, left atrial anteroposterior diameter, left ventricular end-diastolic internal diameter, left ventricular end-systolic internal diameter, septal thickness, left ventricular posterior wall thickness, and the main pulmonary artery diameter (MPAD), were simultaneously recorded.

We depicted the distribution of ascending aortic diameter in the normal population by different gender and age groups (per 10 years). Further, we calculated the proportions of patients with diameters above 40 mm (dilation) and 45 mm (aneurysm), respectively, according to the recommendations of the 2022 American College of Cardiology (ACC)/American Heart Association (AHA) guideline.^6^ Then, we compared the differences in aortic diameter distribution and the incidence of aortic dissection between the HP, BAV, and MFS subgroups and the normal populations, respectively.

### Statistical analysis

Continuous variables were tested for normality distribution using the Kolmogorov-Smirnov test and were expressed as a mean with standard deviation (SD) or median with an interquartile range (IQR). An independent *t*-test was performed for normally distributed variables, or Mann–Whitney *U*-test otherwise. Categorical variables were presented as frequencies with percentages, and analysed by the χ^2^ test or Fisher’s exact test, as appropriate. Pearson test was performed to determine the correlation between aortic diameter and other echocardiographic parameters. R software (version 4.2.1) was employed for data analysis. A two-tailed *P* < 0.05 was considered to indicate a statistically significant difference.

## Results

**Figure 1:**
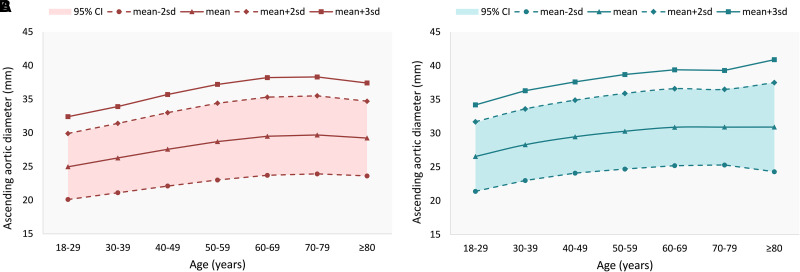
Aortic diameter distribution for normal population: (*A*) aortic diameter reference value in females and (*B*) aortic diameter reference value in females.

*Table 1* provides the baseline characteristics of the normal population (*n* = 86,983, comprising 40 886 men (47%), and 46 097 women (53%). Left ventricular ejection fraction (LVEF) in this cohort was 66.6 ± 4.2% (men vs. women: 66.2 ± 4.2% vs. 66.9 ± 4.3%, *P* < 0.001). The mean value of the ascending aortic diameter was 28.1 ± 3.2 mm, with 27.2 ± 3.1 mm in women and 29.0 ± 3.1 mm in men (*P* < 0.001). The detailed distribution of aortic diameters in men and women is presented in *Table 2* and *Figure 1*. The aortic diameter in men tended to be larger than that in women in each age group (*P* < 0.001) and increased gradually with age (*P* < 0.001). We provide 2 SD (∼5% of the population) and 3 SD (∼1% of the population) as two reference lines for the upper limit of abnormality. *Table 2* can be used as a reference for normal values of the ascending aorta. Most people are supposed to have an aortic diameter within the mean value ± SD. If the diameter is greater than the mean value +2 SD, careful follow-up might be needed. If the diameter is greater than the mean value +3 SD, medical consulting is strongly recommended. Correlation results for aortic diameter and other echocardiographic parameters are shown in Supplementary data online, *Table S1*.

**Table 1:** Demographic data of the normal population

	Total	Female	Male
*n* (%)	86 983	46 097 (53%)	40 886 (47%)
Age (year)	43 (21)	42 (20)	44 (21)
18–29	16 964	8799 (52%)	8165 (48%)
30–39	19 936	11 086 (56%)	8550 (44%)
40–49	21 654	11 594 (54%)	10 060 (46%)
50–59	18 431	9675 (52%)	8756 (48%)
60–69	8534	4175 (49%)	4359 (51%)
70–79	1596	706 (44%)	890 (56%)
≥80	168	106 (37%)	62 (63%)
AAO diameter (mm)	28.1 ± 3.2	27.2 ± 3.1	29.0 ± 3.1
Echocardiography characteristics	86 479	45 886 (53%)	40 593 (47%)
LVEF (%)	66.6 ± 4.2	66.9 ± 4.3	66.2 ± 4.2
LAD (mm)	30.2 ± 3.3	29.6 ± 3.3	30.9 ± 3.3
LVEDD (mm)	43.8 ± 3.6	42.8 ± 3.4	45.0 ± 3.5
LVESD (mm)	26.9 ± 3.1	26.2 ± 2.9	27.7 ± 3.0
IVS (mm)	8.8 ± 1.2	8.4 ± 1.1	9.2 ± 1.1
LVPWT (mm)	8.6 ± 1.2	8.3 ± 1.1	9.0 ± 1.1
MPAD (mm)	21.6 ± 2.4	21.3 ± 2.4	22.0 ± 2.4

AAO, ascending aorta; LVEF, left ventricular ejection fraction; LAD, left atrial diameter; LVEDD, left ventricular end-diastolic diameter; LVESD, left ventricular end-systolic diameter; IVS, interventricular septal thickness; LVPWT, left ventricular posterior wall thickness; MPAD, main pulmonary artery diameter.

**Table 2:** Ascending aortic diameter of the normal population by age and gender

Age	Gender	Mean ± SD (mm)	Mean + 2SD (mm)	Mean + 3SD (mm)
18–29	Female	25.0 ± 2.5	29.9	32.4
	Male	26.6 ± 2.6	31.7	34.2
30–39	Female	26.3 ± 2.6	31.4	33.9
	Male	28.3 ± 2.7	33.6	36.3
40–49	Female	27.6 ± 2.7	33	35.7
	Male	29.5 ± 2.7	34.9	37.6
50–59	Female	28.7 ± 2.8	34.4	37.2
	Male	30.3 ± 2.8	35.9	38.7
60–69	Female	29.5 ± 2.9	35.3	38.2
	Male	30.9 ± 2.8	36.6	39.4
70–79	Female	29.7 ± 2.9	35.5	38.3
	Male	30.9 ± 2.8	36.5	39.3
≥80	Female	29.2 ± 2.8	34.7	37.4
	Male	30.9 ± 3.3	37.5	40.9
*P* value	/	<0.001	/	/

SD, standard deviation.

Further, we established the distribution of ascending aortic diameters in the populations with HP, MFS, and BAV. As can be seen in *Table 3*, the prevalence of HP, MFS, and BAV in the overall population (*n* = 647 087) was estimated to be 7.63% (*n* = 49 374), 0.05% (*n* = 337), and 1.07% (*n* = 6906), respectively. Comparisons of the aortic diameters in populations with HP, MFS, and BAV, with those of the normal population are presented in *Tables 4–6*, respectively. As expected, the aortic diameters of the populations with HP, MFS, and BAV were greater than that of the normal population in each age- and gender-stratified group (*P* < 0.001). As can be observed in *Table 7*, the prevalence of aortic dilation, aneurysm, and dissection were 12.83%, 2.70%, and 4.77% in the HP population, correspondingly, and 43.92%, 35.31%, 26.11% in the MFS population, respectively. High incidences of aortic dilation (37.00%) and aortic aneurysm (16.46%) were observed in the BAV population. However, the incidence of aortic dissection in the BAV population was low (only 0.74%).

**Table 3:** Incidence of HP, Marfan’s syndrome, and BAV among Asian population

	Total	Incidence	Female	Male	*P* value
Overall population	647 087		308 426 (48%)	338 661 (52%)	
Specific population					
HP	49 374	7.63%	13 578 (28%)	35 796 (72%)	<0.001
Marfan’s syndrome	337	0.05%	127 (38%)	210 (62%)	<0.001
BAV	6906	1.07%	2092 (30%)	4814 (70%)	<0.001

**Table 4:** Ascending aortic diameter of the HP population, as compared with the normal group

Age	Gender	Normal	Number	HP	Number	*P* value
		mean ± SD (mm)		mean ± SD (mm)		
18–29	Female	25.0 ± 2.5	8799	30.2 ± 5.4	120	<0.001
	Male	26.6 ± 2.6	8165	31.2 ± 5.4	303	<0.001
30–39	Female	26.3 ± 2.6	11 086	31.2 ± 3.8	230	<0.001
	Male	28.3 ± 2.7	8550	33.7 ± 5.0	1368	<0.001
40–49	Female	27.6 ± 2.7	11 594	33.0 ± 5.1	708	<0.001
	Male	29.5 ± 2.7	10 060	34.7 ± 5.0	4425	<0.001
50–59	Female	28.7 ± 2.8	9675	33.3 ± 4.5	2113	<0.001
	Male	30.3 ± 2.8	8756	35 ± 4.6	8106	<0.001
60–69	Female	29.5 ± 2.9	4175	33.7 ± 4.6	4109	<0.001
	Male	30.9 ± 2.8	4359	35.3 ± 4.6	10 489	<0.001
70–79	Female	29.7 ± 2.9	706	33.7 ± 4.4	3945	<0.001
	Male	30.9 ± 2.8	890	35.3 ± 4.6	7311	<0.001
≥80	Female	29.2 ± 2.8	62	33.4 ± 4.4	2353	<0.001
	Male	30.9 ± 3.3	106	35.2 ± 4.5	3794	<0.001

SD, standard deviation.

**Table 5:** Ascending aortic diameter of the Marfan population, as compared with the normal group

Age	Gender	Normal	Number	MFS	Number	*P* value
		mean ± SD (mm)		mean ± SD (mm)		
18–29	Female	25.0 ± 2.5	8799	36.4 ± 12.0	73	<0.001
	Male	26.6 ± 2.6	8165	46.1 ± 18.3	102	<0.001
30–39	Female	26.3 ± 2.6	11 086	41.5 ± 18.5	29	<0.001
	Male	28.3 ± 2.7	8550	49.9 ± 17.9	61	<0.001
40–49	Female	27.6 ± 2.7	11 594	43.8 ± 12.8	15	<0.001
	Male	29.5 ± 2.7	10 060	41.1 ± 11.0	24	<0.001
50–59	Female	28.7 ± 2.8	9675	46.7 ± 13.2	7	0.0110
	Male	30.3 ± 2.8	8756	54.3 ± 25.9	15	<0.001
60–69	Female	29.5 ± 2.9	4175	38.3 ± 2.8	3	0.0337
	Male	30.9 ± 2.8	4359	47.2 ± 6.9	6	<0.001
70–79	Female	29.7 ± 2.9	706	/	0	/
	Male	30.9 ± 2.8	890	47.5 ± 0.7	2	0.0155
≥80	Female	29.2 ± 2.8	62	/	0	/
	Male	30.9 ± 3.3	106	/	0	/

SD, standard deviation; MFS, Marfan syndrome.

**Table 6:** Ascending aortic diameter of the BAV population, as compared with normal group

Age	Gender	Normal	Number	BAV	Number	*P* value
		mean ± SD (mm)		mean ± SD (mm)		
18–29	Female	25.0 ± 2.5	8799	31.3 ± 5.8	384	<0.001
	Male	26.6 ± 2.6	8165	32.4 ± 7.0	584	<0.001
30–39	Female	26.3 ± 2.6	11 086	33.9 ± 6.9	293	<0.001
	Male	28.3 ± 2.7	8550	35.7 ± 6.4	703	<0.001
40–49	Female	27.6 ± 2.7	11 594	36.8 ± 8.8	278	<0.001
	Male	29.5 ± 2.7	10 060	38.1 ± 6.8	993	<0.001
50–59	Female	28.7 ± 2.8	9675	38.2 ± 6.8	480	<0.001
	Male	30.3 ± 2.8	8756	40.2 ± 7.1	1105	<0.001
60–69	Female	29.5 ± 2.9	4175	39.4 ± 6.2	454	<0.001
	Male	30.9 ± 2.8	4359	39.9 ± 6.7	1020	<0.001
70–79	Female	29.7 ± 2.9	706	39.8 ± 6.3	174	<0.001
	Male	30.9 ± 2.8	890	40.2 ± 6.6	361	<0.001
≥80	Female	29.2 ± 2.8	62	38.8 ± 6.4	29	<0.001
	Male	30.9 ± 3.3	106	39.5 ± 7.3	48	<0.001

SD, standard deviation; BAV, bicuspid aortic valve.

**Table 7:** The prevalence of aortic dilation, aneurysm, and aortic dissection in population with HP, Marfan’s syndrome, and BAV

	Aortic dilation (≥40 mm)			Aneurysm (≥45 mm)			Aortic dissection		
	Total	Female	Male	Total	Female	Male	Total	Female	Male
Overall population	29 981 (4.63%)	8011 (2.6%)	21 970 (6.49%)	6885 (1.06%)	1829 (0.59%)	5056 (1.49%)	5039 (0.78%)	850 (0.28%）	4189 (1.24%)
Specific population									
HP	6353 (12.83%)	1112 (8.19%)	5241 (14.64%)	1334 (2.70%)	248 (1.83%)	1086 (3.03%)	2354 (4.77%)	292 (2.15%)	2062 (5.76%)
Marfan’s syndrome	148 (43.92%)	37 (29.13%)	210 (52.86%)	119 (35.31%)	26 (20.47%)	93 (44.29%)	88 (26.11%)	24 (18.90%)	64 (30.48%）
Bicuspid aortic valve	2555 (37.00%)	688 (32.89%)	4814 (38.78%)	1137 (16.46%)	285 (13.62%)	852 (17.70%)	51 (0.74%)	7 (0.33%)	44 (0.91%)

**Figure 2:**
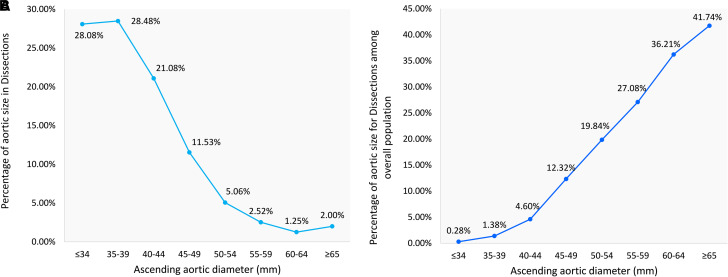
Aortic paradox explained: (*A*) aortic dissection size distribution and (*B*) aortic dissection size distribution among the overall population.

The aortic diameter distribution of type A aortic dissection is presented in *Figure 2* and Supplementary data online, *Table S2*. The percentage of the aortic dissection in the subgroup of patients with aortic dissection (numerator) seemed to be reduced with the increase in the aortic diameter (28.08% of the aortic dissection diameter < 35 mm, and 2.00% of the aortic dissection diameter ≥65 mm). Hence, most cases of aortic dissection occurred with an aortic diameter of less than 55 mm. However, in the overall population (denominator), the incidence of aortic dissection increased significantly with the rise in the aortic diameter (aortic dissection occurred in 0.28% of the population with an aortic diameter <35 mm and in 41.71% of the population with an aortic diameter ≥ 65 mm).

## Discussion

In this study, we investigated the distribution of ascending aortic diameters in the normal population based on age and gender. The diameter distribution and dissection rates in patients with HP, BAV, and MFS were also analysed and compared with those in the normal population. The major findings can be summarized as follows. First, the diameter of the ascending aorta is closely related to age and gender: the diameter increased with age and was larger in men than in women. Second, the aortic diameter in Asian populations is smaller than in Western populations, and the guidelines need to consider racial differences. Third, the ‘aortic paradox’ was addressed: although most aortic dissections occurred at an aortic diameter of <55 mm, the incidence of aortic dissection was exponentially positively correlated with the aortic diameter in the overall population. Fourth, BAV bears a high rate of aortic dilation, but a low incidence of aortic dissection, supporting a more conservative treatment strategy.

Thoracic aortic disease is dominated by TAA, which is defined as an aortic diameter greater than 1.5 times the normal aorta or exceeding the mean value by 2 SD. Aortic dilation is also an often-mentioned concept, with some scholars proposing an aortic diameter of 3.5 cm or more as dilation,^7^ and others proposing 4 cm. The 2022 AHA/ACC aortic guideline^6^ stated the criteria for aortic dilation as 4 cm and for aortic aneurysm as 4.5 cm. Currently, these concepts have not been fully harmonized. One of the big hurdles is our lack of evidence-based medical data for normal aortic diameters established in large samples, while we can recognize abnormalities only by determining the normal diameter. This study included 698 795 Asian individuals and aimed to provide reference values for normal and abnormal aortic diameters categorized by gender for all age groups. The present findings are expected to confer important references for the management of aortic disease and patient education. Our study, together with previous studies,^8,9^ revealed strong correlations between aortic diameter and gender and age. The mean aortic diameters in men and women were 29.0 ± 3.1 and 27.2 ± 3.1 mm, respectively (*P* < 0.001). The results of this study showed that the upper limit of the normal value was 34.2 mm for men aged 18–29 years and 40.9 mm for men aged 80 or older. For convenience, 40 mm may be regarded as the normal upper limit for the overall population. However, as we recommended, the aortic diameter indexed by gender and age should be considered whenever possible for preciseness.

The mean aortic diameter of the normal population was 28.1 ± 3.2 mm in our study, which is close to previous studies in Asian populations. Li *et al*.^10^ showed a mean diameter of 28.9 ± 4.6 mm in the ascending aorta using magnetic resonance imaging (MRI). Lee *et al.*^11^ found that the mean aortic diameter in the Korean population was 29.9 ± 5.7 mm. The findings of the aforementioned studies are in contrast with the data obtained for Western populations. A study by Wolak *et al.*^12^ including 2952 patients established that the mean aortic diameter in the American population was 33 ± 4 mm, much greater than the one determined in the present investigation. Similarly, the results of Ayoub *et al.*^13^ showed a mean aortic diameter of 31.7 ± 4.1 mm in the American population. Interestingly, Wang *et al.*^14^ found that the clinical presentations of type A aortic dissection in China also differed significantly from those of Western populations. The findings of these studies indicate that it is necessary to probe into the possible influence of race on aortic diseases, especially the natural history studies, which are pivotal in deciding surgical indication. The primary data currently available are from the Elefteriades-led Yale Aortic Center database, which includes patients mainly from the New England region of the United States. The evidence generated by this database is being promoted worldwide. Nevertheless, we should be alert to the fact that current guidelines give minimal consideration to racial differences. There had been gender-based natural history studies for descending aorta,^15^ but regretfully, ascending TAA natural history studies based on gender or age are poorly understood. It is important that more attention should be focused on this aspect of research work, as aortic diameter is closely related to gender and age.

The present study is a diameter-based investigation of ascending aorta. Recently there have been some questions about the aortic diameter as an indication of TAA. Elefteriades first elucidated the natural history of TAA in 1997^16^ and established a certain cut-off of aortic diameter as a surgical indication for TAA through a series of follow-up studies.^17,18^ A dramatically increased risk of AAE was observed in patients with an aortic diameter of more than 60 mm; therefore, the aortic diameter of 55 mm was considered as the threshold, which was officially recommended by the 2010 AHA and 2014 European Society of Cardiology (ESC) guidelines.^19^ However, the International Registry of Acute Aortic Dissection (IRAD) data showed that out of 591 patients with type A aortic dissection, a total number of 349 (59%) had an aortic diameter less than 55 mm and 229 (40%) less than 50 mm.^20^ Accordingly, Pape *et al.* concluded that an aortic diameter of 55 mm is not an appropriate indication of aortic aneurysm, as a large proportion of patients with impending aortic dissection might be neglected due to this threshold. Elefteriades *et al.* argued that the population with a small aortic diameter (denominator) is large; thus, even if the incidence of aortic dissection in this group is very low, its absolute number is still large. This phenomenon is also known as the ‘aortic paradox’.^21^ There is no strong data to justify this phenomenon. We evidenced that if we were to focus only on the aortic diameter distribution in patients with aortic dissection, the results would be the same as those available in IRAD.^20^ Specifically, the aortic diameters of most patients with aortic dissection were less than 50–55 mm, and the curve showed that the percentage of aortic dissection decreased with the increment of the aortic diameter. We found that 28.08% of the aortic dissection occurred at a diameter of <35 mm, whereas 2.00% of it occurred at a diameter of ≥65 mm. However, the trend was totally reversed after we calculated the incidence of aortic dissection (numerator) in the overall population (denominator). More specifically, the incidence of aortic dissection increased sharply with the rise in the aortic diameter. The incidence of aortic dissection in the population with an aortic diameter of <35 mm was only 0.28%, compared to 41.74% in the population with an aortic diameter of ≥65 mm.

Another important finding of this study is the strong contrast between the high percentage of aortic dilation and the low incidence of aortic dissection in BAV. Our study showed that the BAV population had a significantly higher incidence of aortic dilation and aneurysm compared with the normal population, but the incidence of aortic dissection was slightly lower. This finding is contradictory to the ones of some previous studies. For a long time, BAV was considered an important risk factor for aortic dissection, and some authors even regarded BAV as a connective tissue disease similar to MFS.^22^ In this regard, Russo *et al.* recommended prophylactic aortic replacement for BAV patients with mildly dilated aorta.^23^ The 2006 AHA/ACC guideline.^24^ also recommended prophylactic aortic replacements for BAV patients undergoing aortic valve replacement with the same criteria used for MFS (45 mm). Surgical indication, whether aggressive or conservative, will have a certain impact, considering the incidence of BAV in the whole population is as high as 1–2%.^25,26^ In our study, the incidences of aortic dilation for the normal population, BAV, and MFS were 4.63%, 37.00%, and 43.92%, respectively. However, this trend was not seen in the incidence of aortic dissection, with an incidence of 0.74% in BAV and 26.11% in MFS. BAV seems to be much safer compared with MFS. Zafar and Wu (the first author of the present study) *et al.*^27^ found that despite the faster aortic growth rate in BAV, the 10-year complication-free survival rate is even higher in patients with BAV than in patients with tricuspid. Currently, the 2022 AHA/ACC guideline recommended a threshold of 55 mm as the surgical indication for patients with BAV, which is even higher than that of patients with tricuspid aortic valve (50 mm). Our data also support the implementation of a more conservative therapeutic strategy for patients with BAV.

### Study limitations

The limitation of this study should be acknowledged. First, this work was based on an Asian population and thus, as mentioned earlier, the extrapolation of the findings needs further justification given the racial differences. Second, the aortic diameters were not indexed by height and weight. Unfortunately, our ultrasound database did not record demographic data. This may not be a practical issue as aortic diameter remains the most commonly used parameter given its reliability and availability.

## Conclusions

(i) The diameter of the ascending aorta is closely related to age and gender, with the diameter increasing with age and being larger in men than in women; (ii) The aortic diameter in Asian populations is smaller than in Western populations, and the guidelines need to consider the racial differences; (iii) The ‘aortic paradox’ was addressed: although most aortic dissections occurred at an aortic diameter of less than 55 mm, the incidence of aortic dissection is exponentially positively correlated with the aortic diameter in the overall population; (iv) BAV bears a high rate of aortic dilation, but a low incidence of aortic dissection, supporting a more conservative treatment strategy.

## Supplementary Material

qyad019_Supplementary_Data

## Data Availability

The data that support the findings of this study are available on request from the corresponding author. The data are not publicly available due to the policy of our institution.
