# Supplementary material for: Aortic size distribution among normal, hypertension, bicuspid, and Marfan populations
Source: Eur Heart J Imaging Methods Pract. 2023 Aug 30;1(2):qyad019. doi: 10.1093/ehjimp/qyad019 (PMC11195692; doi:10.1093/ehjimp/qyad019)

**Supplemental Table 1.** Correlation between echocardiographic characteristics and ascending aortic diameter.

| **Echocardiography characteristics** | **R** | **P** |
| --- | --- | --- |
| LVEF (%) | -0.06 | ＜0.001 |
| LAD (mm) | 0.42 | ＜0.001 |
| LVEDD (mm) | 0.23 | ＜0.001 |
| LVESD (mm) | 0.18 | ＜0.001 |
| IVS (mm) | 0.39 | ＜0.001 |
| LVPWT (mm) | 0.38 | ＜0.001 |
| MPAD (mm) | 0.34 | ＜0.001 |

LVEF: left ventricular ejection fraction; LAD: left atrial diameter;

LVEDD: left ventricular end-diastolic diameter; LVESD: left ventricular end-systolic diameter;

IVS: interventricular septal thickness; LVPWT: left ventricular posterior wall thickness;

MPAD: main pulmonary artery diameter.

**Supplemental Table 2.** Risk of aortic dissection in each diameter group.

| **Aortic ascending diameter (mm)** | **<35** | **35-39** | **40-44** | **45-49** | **50-54** | **55-59** | **60-65** | **≥65** |
| --- | --- | --- | --- | --- | --- | --- | --- | --- |
| Overall population (n) | 513470 | 103636 | 23096 | 4715 | 1285 | 469 | 174 | 242 |
| Aortic dissection (n) | 1415 | 1435 | 1062 | 581 | 255 | 127 | 63 | 101 |
| Percentage of aortic size in dissections (%) | 28.08% | 28.48% | 21.08% | 11.53% | 5.06% | 2.52% | 1.25% | 2.00% |
| Percentage of aortic size for dissections among overall population (%) | 0.28% | 1.38% | 4.60% | 12.32% | 19.84% | 27.08% | 36.21% | 41.74% |

**Supplemental Figure 1**. The detailed data screening process


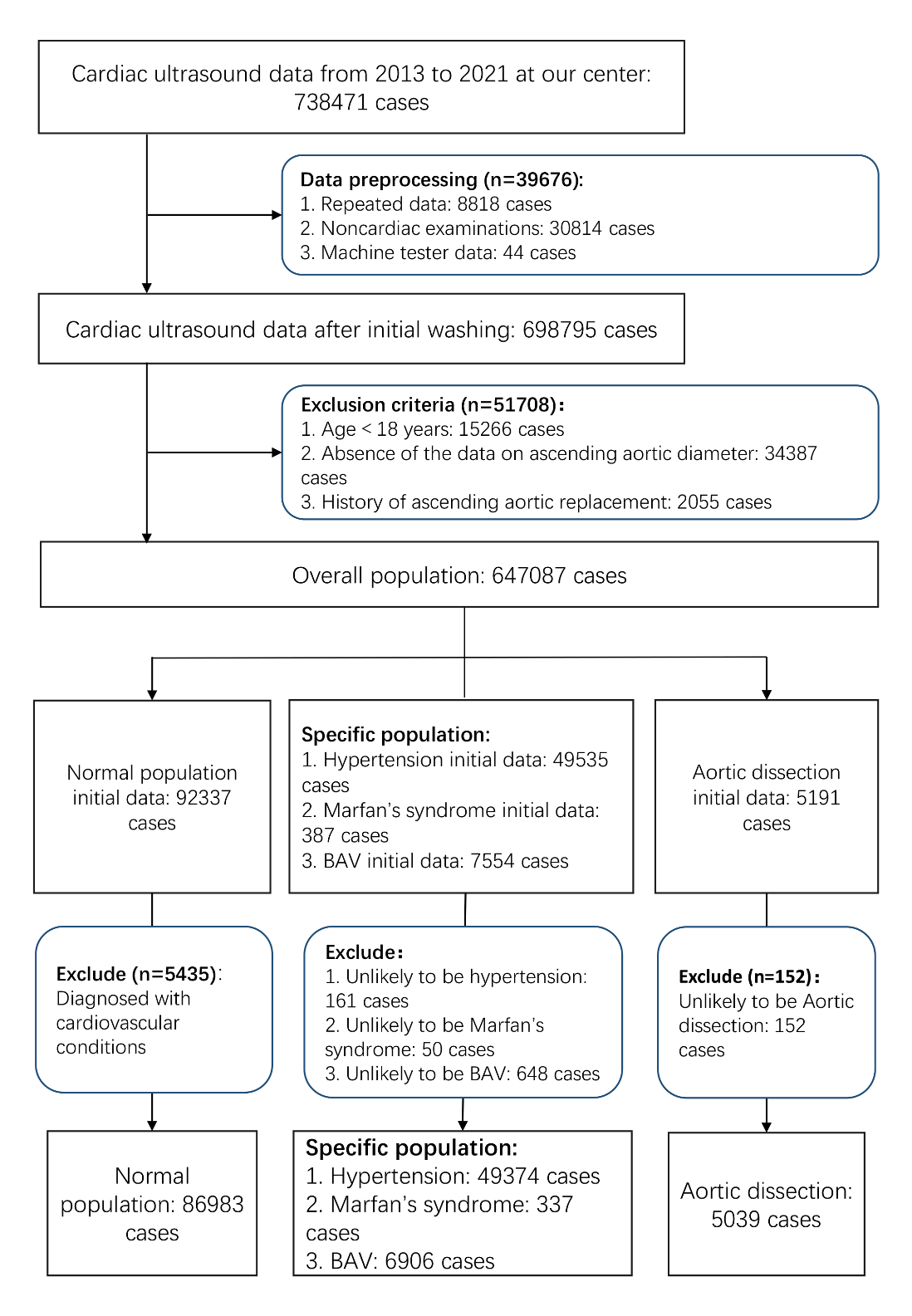

Supplement: qyad019_Supplementary_Data [file qyad019_Supplementary_Data.docx]
